# DNA methylation in adults and during development of the self‐fertilizing mangrove rivulus, *Kryptolebias marmoratus*

**DOI:** 10.1002/ece3.4141

**Published:** 2018-05-15

**Authors:** Alexandre Fellous, Tiphaine Labed‐Veydert, Mélodie Locrel, Anne‐Sophie Voisin, Ryan L. Earley, Frederic Silvestre

**Affiliations:** ^1^ Laboratory of Evolutionary and Adaptive Physiology Institute of Life, Earth and Environment University of Namur Namur Belgium; ^2^ Department of Biological Sciences University of Alabama Tuscaloosa Alabama USA

**Keywords:** development, DNA methylation, *Kryptolebias marmoratus*, LUMA, reprogramming, self‐fertilization

## Abstract

In addition to genetic variation, epigenetic mechanisms such as DNA methylation might make important contributions to heritable phenotypic diversity in populations. However, it is often difficult to disentangle the contributions of genetic and epigenetic variation to phenotypic diversity. Here, we investigated global DNA methylation and mRNA expression of the methylation‐associated enzymes during embryonic development and in adult tissues of one natural isogenic lineage of mangrove rivulus fish, *Kryptolebias marmoratus*. Being the best‐known self‐fertilizing hermaphroditic vertebrate affords the opportunity to work with genetically identical individuals to examine, explicitly, the phenotypic effects of epigenetic variance. Using the LUminometric Methylation Assay (LUMA), we described variable global DNA methylation at CpG sites in adult tissues, which differed significantly between hermaphrodite ovotestes and male testes (79.6% and 87.2%, respectively). After fertilization, an immediate decrease in DNA methylation occurred to 15.8% in gastrula followed by re‐establishment to 70.0% by stage 26 (liver formation). Compared to zebrafish, at the same embryonic stages, this reprogramming event seems later, deeper, and longer. Furthermore, genes putatively encoding DNA methyltransferases (DNMTs), Ten‐Eleven Translocation (TET), and MeCP2 proteins showed specific regulation in adult gonad and brain, and also during early embryogenesis. Their conserved domains and expression profiles suggest that these proteins play important roles during reproduction and development. This study raises questions about mangrove rivulus’ peculiar reprogramming period in terms of epigenetic transmission and physiological adaptation of individuals to highly variable environments. In accordance with the general‐purpose genotype model, epigenetic mechanisms might allow for the expression of diverse phenotypes among genetically identical individuals. Such phenotypes might help to overcome environmental challenges, making the mangrove rivulus a valuable vertebrate model for ecological epigenetic studies. The mangrove rivulus, Kryptolebias marmoratus, is the best‐known self‐fertilizing hermaphroditic vertebrate that allows to work with genetically identical individuals to examine, explicitly, the phenotypic effects of epigenetic variance. The reprogramming event is later, more dramatic and longer than in other described vertebrates. High evolutionary conservation and expression patterns of DNMT, TET, and MeCP2 proteins in *K. marmoratus* suggest biological roles for each member in gametogenesis and development.

## 1 INTRODUCTION

The Modern Evolutionary Synthesis assumes that genetic diversity is the only source of heritable variation in natural populations. Random mutations can explain this genetic variability and promote evolution by means of natural selection (Mayer & Provine, 1998). However, there is an increasing body of evidence showing that genetic variation is not the only source of heritable phenotypic diversity and that epigenetic variation can also contribute to heritable changes within populations, and consequently drive rapid evolution (Bossdorf, Richards, & Pigliucci, 2008; Skinner, 2015).

Epigenetics has been defined as the study of mitotically and/or meiotically heritable changes in gene expression that cannot be explained by changes in DNA sequence (Fincham, 1997). It includes noncoding RNA activity, histone modification, and DNA methylation, the latter being the most studied mechanism. In eukaryotes, DNA methylation mostly refers to the transfer of a methyl group to position 5 of cytosine residues to form 5‐Methylcytosine (5 mC), mainly in a CpG dinucleotide context (Feng et al., 2010; Law & Jacobsen, 2010; Lister & Ecker, 2009). This reaction is catalyzed by a group of enzymes, the DNA (cytosine‐5)‐methyltransferases (DNMTs) (Hsieh, 1994; Kass, Landsberger, & Wolffe, 1997), with three conserved proteins (DNMT1, DNMT3a, and DNMT3b) being characterized in vertebrates (Campos, Valente, & Fernandes, 2012; Goll & Bestor, 2005; Li, Bestor, & Jaenisch, 1992; Okano, Bell, Haber, & Li, 1999). DNMT1 is the maintenance enzyme, which restores DNA methylation after replication, whereas DNMT3a and DNMT3b catalyze *de novo* methylation (Li et al., 1992; Okano et al., 1999). Typically associated with transcriptional repression, DNA methylation may also influence compaction of chromatin and therefore transcriptional activity of a genomic region by interfering with DNA binding of the transcriptional machinery directly or via a methyl‐DNA‐binding domain (MBD) protein such as MeCP2 (Liang et al., 2011; Lindeman et al., 2010). In addition to 5 mC, another modification, DNA 5‐hydroxymethylation (5 hmC), recently characterized in vertebrate genomes (Zhao & Chen, 2013), is catalyzed by the Ten‐Eleven Translocation (TET) enzymes via oxidation of 5 mC marks (Tahiliani et al., 2009). However, the precise relationships between 5 mC and 5 hmC still remain to be established (Kamstra, Alestrm, Kooter, & Legler, 2015; Kamstra, Løken, Aleström, & Legler, 2015). Despite the conserved role of 5 mC in gene silencing, DNA methylation levels vary significantly among eukaryotes (Feng et al., 2010) from very limited methylation in ecdysozoan protostomes (Gowher, Leismann, & Jeltsch, 2000) to CpG sites generally highly methylated in vertebrates (Head, 2014). 5 mC is of great biological significance because of its role in genome stability (Maloisel & Rossignol, 1998), imprinting (Bell & Felsenfeld, 2000), X‐chromosome inactivation (Csankovszki, Nagy, & Jaenisch, 2001), gene expression and development (Jaenisch & Bird, 2003). One of the most significant features of DNA methylation is that it can be mitotically and/or meiotically heritable (Wu & Morris, 2001) and, in some circumstances, can be transgenerationally inherited (Chong & Whitelaw, 2004; Jablonka & Raz, 2009; Richards, 2006). Such Transgenerational Epigenetic Inheritance (TEI) is of crucial importance to understand the role of epigenetics in evolution even if most epigenetic mutations (epimutations) have been reported to be neutral or deleterious (Heard & Martienssen, 2014).

One of the main obstacles that epimutations must overcome to be inherited is DNA methylation reprogramming, which occurs twice, once in the germline and once in the early embryo. Reprogramming can be seen as an erasure of epigenetic marks required for correct development of the embryo and establishment of DNA methylation patterns in the new individual (Monk, Boubelik, & Lehnert, 1987). In the mouse, the two parental genomes are actively and passively demethylated until early cleavage states before being remethylated with the help of DNMT3a and DNMT3b enzymes (Edwards, Yarychkivska, Boulard, & Bestor, 2017). In other vertebrates, little is known about reprogramming. In fish, the only studies so far have been conducted in zebrafish, *Danio rerio* (Fang, Thornton, Scheffler, & Willett, 2013; Mhanni & McGowan, 2004), and it has been shown that DNA methylation reprogramming represents a highly critical window sensitive to environmental stress (Dorts et al., 2016; Martin, Laforest, Akimenko, & Ekker, 1999). Deciphering TEI, environmental cues and reprogramming are consequently important to understand the putative role of epigenetics in rapid adaptation and evolution.

**Figure 1:**
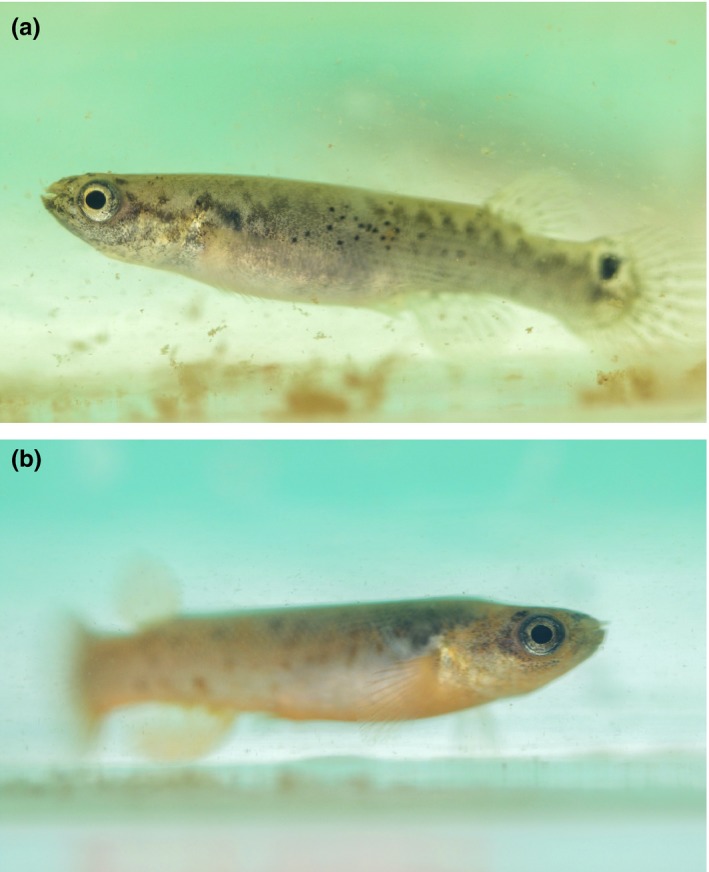
Different phenotypes of *Kryptolebias marmoratus* obtained after self‐fertilization of one hermaphrodite. (a) Hermaphrodite; (b) Male. (Photograph credit: Alexandre Fellous)

Another difficulty that must be overcome to examine the role of epigenetic variation in evolution is the fact that we can rarely exclude DNA sequence differences among individuals to explain heritability of phenotypes. Consequently, a deep understanding of the role of epigenetic variation in evolution can only be achieved in individuals that are genetically identical but exhibit a range of heritable phenotypes in nature (Heard & Martienssen, 2014). In vertebrates, such model species are scarce and we propose here to characterize global CpG DNA methylation, as well as expression of DNA methylation and demethylation enzyme genes in mangrove rivulus fish, *Kryptolebias marmoratus*. Together with its sister species, *Kryptolebias hermaphroditus,* mangrove rivulus is the only known vertebrate able to self‐fertilize (Costa, 2011; Harrington, 1961; Tatarenkov, Lima, Taylor, & Avise, 2009). In nature, hermaphrodites coexist with a low proportion of males (mostly fewer than 5%) (Avise & Tatarenkov, 2015; Mackiewicz, Tatarenkov, Taylor, Turner, & Avise, 2006; Mackiewicz, Tatarenkov, Turner, & Avise, 2006; Mackiewicz, Tatarenkov, Perry, et al., 2006), but no females, which constitutes a rare androdioecious mixed reproductive system (Avise & Tatarenkov, 2012; Weeks, Crosser, Bennett, Gray, & Zucker, 2000). Given the ability of hermaphrodites to lay unfertilized eggs, outcrossing with males is possible but less frequent than selfing (Mackiewicz, Tatarenkov, Taylor, et al., 2006; Mackiewicz, Tatarenkov, Turner, et al., 2006; Mackiewicz, Tatarenkov, Perry, et al., 2006). However, most of the time, they self‐fertilize by internal fecundation and many generations of exclusive selfing give rise to natural isogenic strains (Mackiewicz, Tatarenkov, Taylor, et al., 2006; Mackiewicz, Tatarenkov, Turner, et al., 2006; Mackiewicz, Tatarenkov, Perry, et al., 2006; Tatarenkov, Earley, Taylor, & Avise, 2012). The mangrove rivulus occupies a large geographic area including the Florida peninsula and associated Keys, Bahamas islands, Central America, and possibly portions of Cuba and Puerto Rico (Avise & Tatarenkov, 2015; Tatarenkov, Lima, & Avise, 2011; Taylor, 2000). Closely affiliated to the red mangrove *Rhizophora mangle* forests with a wet‐dry seasonal alternation and semidiurnal tides with very high tides in fall and very low tides in spring/summer, mangrove rivulus exhibit numerous adaptations to living in microhabitats, such as crab burrows or ephemeral pools, that show considerable variation in salinity, oxygen, ammonia, and temperature (Ellison et al., 2012). Finally, this species exhibits high phenotypic plasticity (Figure 1) within and between isogenic lineages (Taylor, 2000) in life history traits (fecundity and sex ratio) (Grageda, Sakakura, Minamimoto, & Hagiwara, 2005), in embryogenesis (diapause) (Mesak, Tatarenkov, & Avise, 2015) and in sexual phenotype. Embryos of *K*.* marmoratus* reared at low temperature (18°C) can develop directly as primary males; also, adult hermaphrodites may undergo sex change to become secondary males (Earley, Hanninen, Fuller, Garcia, & Lee, 2012; Harrington, 1961; Turner, Fisher, Taylor, Davis, & Jarrett, 2006). Thus, Ellison et al. (2015) suggested that natural variation in self‐fertilization rates among populations might be explained through epigenetic regulation (DNA methylation) of sex ratios.

Investigating global CpG DNA methylation and associated enzyme gene expression profiles in mangrove rivulus will provide a basis to understand links between epigenetic mechanisms, phenotypic plasticity, and environmental cues, as well as the potential of TEI, a prerequisite to understand the putative role of epigenetics in evolution. Together with its biological characteristics, mangrove rivulus is a very promising model to address key questions in ecotoxicology, behavior, physiology, and evolutionary biology (Kelley et al., 2012; Voisin, Fellous, Earley, & Silvestre, 2016), and it allows researchers to distinguish epigenetic from genetic contributions to phenotypic plasticity. In this study, we measured global CpG methylation levels in genomic DNA throughout early development, i.e.from fertilized eggs to hatching, in juveniles and in adult brain, liver, gills, gonads, skin, and muscle, using the LUminometric Methylation Assay (LUMA). The availability of public transcriptomic databases and of the mangrove rivulus genome (Kelley et al., 2016) led to identification and molecular characterization of sequences putatively encoding DNMT, TET, and MeCP2 orthologues in this species.

## 2 METHODS

### 2.1 Ethic statement

All rivulus husbandry and experimental procedures were performed in accordance with the Belgian animal protection standards and were approved by the University of Namur Local research Ethics Committee (UN KE 16/258). The agreement number of the laboratory for fish experiments is the LA1900048.

### 2.2 Animals

Adults rivulus from DC4 lineage were sampled in 2010 from the Florida Keys (Dove Creek; Tavernier, Florida; N25°01′45.64″, W080°29′49.24″). This isogenic lineage is homozygous at all loci tested by microsatellite analysis (Mackiewicz, Tatarenkov, Taylor, et al., 2006; Mackiewicz, Tatarenkov, Turner, et al., 2006; Mackiewicz, Tatarenkov, Perry, et al., 2006) and is maintained in both Silvestre and Earley laboratories. Living rivulus were kept individually in 500 mL plastic aquaria in a controlled environment (26 ± 1°C; 25 ppt salinity; 12 hr: 12 hr light/dark photoperiod) and were fed with living brine shrimp (*Artemia*) nauplii. Reconstituted seawater (25 ppt) was produced by mixing marine salts (Instant Ocean salt) and demineralized water. Spawning cottons were added to the tanks at sexual maturity (100 days (Cole, Noakes, & Aug, 1997)) to provide a substrate for oviposition.

### 2.3 Sampling

Experimental fish (adults; *n* = 7) and juveniles (*n* = 12 per time point: 1, 2, 6, 10, 12, 15, and 20 days posthatching) from the DC4 lineage were sacrificed in 4°C seawater, decapitated, and then adult tissues (liver, gonads, brain, muscle, gills, and skin) were collected. The entire body of the juveniles was used. Sampling of developmental stages (at least 100 eggs per stage) was conducted by microscopic observation of morphological and mobility criteria as previously described (Mourabit, Edenbrow, Croft, & Kudoh, 2011) from fertilized eggs to stage 32 (hatching) (Figure S1). However, due to the internal fertilization, it was difficult to assess exact fertilization time (Sakakura, Soyano, Noakes, & Hagiwara, 2006).

### 2.4 Imaging

All photographs were taken using a Nikon Digital Camera USB3 1/2.5 15 IM/SEC mounted on a Nikon SMZ1270 Stereomicroscope using the NIS‐D software. For photographic purposes, rotations of the embryos were maintained using dissecting forceps within the camera frame.

### 2.5 Global DNA methylation quantification

Genomic DNA from embryos, juveniles, and adult tissues was purified using affinity chromatography (Macherey‐Nagel 740952 and 740901, Germany) according to manufacturer's protocol. DNA samples were treated with RNase A (Macherey‐Nagel, 740505, Germany) to remove RNA contamination and provide accurate quantification of DNA, especially for transcriptionally active tissues such as liver (Head, Mittal, & Basu, 2014). DNA concentration and sample quality were assessed by spectrophotometry (NanoDrop 2000c Spectrophotometer, Thermo Scientific) and 1.5% agarose gel electrophoresis. Samples that were not of high molecular weight, meaning that DNA is degraded, were rejected. Quantification of global DNA methylation was examined using LUminometric Methylation Assay (LUMA) based on the original method (Head et al., 2014; Karimi et al., 2006; Richard Pilsner et al., 2010). Each 20 μl reaction contained 600 ng of DNA, 2 μl of 10X Tango Buffer (New England BioLabs), 0.5 μl of *MunI* (input DNA), and 0.5 μl of either *HpaII* or *MspI* (methylation sensitive and insensitive, respectively); all enzymes were supplied by New England Biolabs. About 15 μl of annealing buffer (Qiagen) was added to each sample, and 30 μl of the resulting product was aliquoted into a pyrosequencing plate. Plates were loaded into a Pyromark Q24 (Qiagen) and the dispensation order for nucleotides was GTGTCACATGTGTG. Methylation values were calculated according to the formula:

$$\begin{array}{cc} \%\text{of Methylation}= & 100*\left(\right.1-\left(\left(\text{HpaII}\left(G\right)/\right.\right.\\ & \left.\left.\text{MunI}\left(T\right)\right)/\left(\text{MspI}\left(G\right)/\left(\text{MunI}\left(T\right)\right)\right)\right) \end{array}$$

G represents the peak height for *HpaII* (cuts the recognition site when unmethylated) or *MspI* (insensitive to methylation status of the recognition site), and T represents the peak height for *MunI* (input DNA). Theoretically, as data are normalized to the *MunI* peak, variable DNA input should not influence the level of DNA methylation. To avoid underestimating methylation values, pyrograms that exhibited quality control corresponding to signal‐to‐noise <6 (ratio between *HpaII* (G) peak height and background) and/or nonspecific peaks (evidence of DNA degradation) were rejected (Head et al., 2014).

### 2.6 Identification of putative DNMTs, MeCP2, and TETs orthologues

The rivulus genome, GenBank database, and public transcriptomic database were screened. Homology‐producing sequences were named *DNMT* (*DNMT1*,* DNMT3av1*,* DNMT3av2*,* DNMT3bv1*,* DNMT3bv2*,* DNMT3bv3*), *TET* (*TET1*,* TET2*,* TET3v1*,* TET3v2*), and *MeCP2* (GenBank accession number, Table S1), respectively, with respect to the name of their most similar sequence at the time of the study. Conserved domains were identified on the National Center for Biotechnology Information (NCBI)'s Conserved Domain Database (CDD) website and domain nomenclature followed in Humans was used. Gene_Name_***v***_x indicates Isoforms of each gene.

### 2.7 Phylogenetic analyses

Sequences encoding DNMT, TET, and MeCP2 proteins from various organisms were obtained through a BLASTX search against the NCBI genome server and were aligned with the Muscle algorithm (Edgar, 2004) using MEGA software version 7 (Kumar, Stecher, & Tamura, 2016). Plants and microorganisms were not included in the analysis due to low homologies, which would have compromised reliability of alignments. Alignment corrections for gaps were performed on Gblocks software (Talavera & Castresana, 2007). Phylogenetic analyses were performed by maximum likelihood (Bootstrap method: 500 bootstraps, complete deletion of gaps) using MEGA software version 7 (Kumar et al., 2016). Results were compared by Neighbor‐joining and minimum evolution methods (Bootstrap method: 500 bootstraps, complete deletion of gaps). Phylogenetic analyses of mangrove rivulus DNMT/TET gene products were performed based on alignments between mangrove rivulus sequences and their putative cognate orthologues in various animal genomes using the gap missing protein sequence.

### 2.8 Molecular characterization

The actual presence of characterized mRNAs was confirmed by PCR amplification using the Phusion High‐Fidelity DNA Polymerase (NEW ENGLAND Biolabs) from cDNA of mangrove rivulus embryos at different stages and adult tissues in a 20 μl reaction volume containing 200 μM DNTPs, 0.5 μM forward primer, 0.5 μM reverse primer, 1 unit of Phusion DNA polymerase and the appropriate buffer in nuclease‐free water. Samples were submitted to the following cycling parameters (98°C, 30 s; 30 cycles of 98°C, 10 s; annealing temperature, 30 s; 72°C, 30 s per kb followed by a final extension of 72°C, 10 min) (see Supplementary data for primer sequences and annealing temperatures (Table S2). Obtained sequences were validated *in silico*.

### 2.9 Reverse transcription quantitative PCR (RT‐qPCR)

RT‐qPCR was performed as previously described (Riviere, Fellous, Franco, Bernay, & Favrel, 2011). Briefly, samples were extracted using affinity chromatography (Nucleospin RNA II kit, Macherey‐Nagel). After digestion of genomic DNA with 1 U RQ1 DNAse (Promega) for 30 min to prevent genomic DNA contamination, 250 ng of total RNA was reverse transcribed using 200 U of M‐MLV RT (Promega) and 100 ng random hexamers. Resulting cDNAs were diluted and the equivalent amount of 5 ng of starting RNA was assayed for expression of each *DNMT*,* TET,* and *MeCP2* family member using *ß‐actin* and *18S RNA* as reference genes. SYBR‐green quantitative PCR was conducted on a StepOnePlus Real‐Time PCR System (Applied Biosystems). GoTaq qPCR master mix (Promega) was used in 45 cycle (95°C, 15 s; 58°C, 15 s) reactions. The primers used are listed in (Table S3). A melting curve and an end‐point agarose gel electrophoresis followed by SYBR safe (Thermofisher) staining were used to check for accurate amplification of the target amplicon. Parallel amplification of a reference gene was carried out to normalize expression data of DNMTs and TETs transcripts. Relative expression of DNMTs and TETs was calculated for one copy of the reference gene using the following formula: *N* = 2^(CtRefgene–CtTargetgene)^. Water was used instead of cDNA as a negative control for amplification, and DNAse untreated cDNA was used to check for absence of genomic DNA contamination. All samples were analyzed in triplicate to establish the mRNA expression profile of mangrove rivulus *DNMTs*,* TETs*, and *MeCP2*.

### 2.10 Statistical analysis

All results are presented as mean ± SEM (Standard Error of Mean) of at least three biological/technical replicates. A one‐way ANOVA (*p* < 0.05) followed by Tukey's post hoc test was performed on the level of CpG global DNA methylation of the developmental stages and juveniles. A two‐way ANOVA (*p* < 0.05) followed by Sidak's post hoc test was performed on the level of CpG global DNA methylation of adult tissues. A one‐way ANOVA (*p* < 0.05) followed by Tukey's post hoc test was performed on mRNA expression levels of *DNMT* (*DNMT1*,* DNMT3av1*,* DNMT3av2*,* DNMT3bv1*,* DNMT3bv2*,* DNMT3bv3*), *MeCP2,* and *TET* (*TET2*,* TET3v1*,* TET3v2*) during development. A two‐way ANOVA (*p* <0.05) followed by Sidak's post hoc test was performed on mRNA expression level of *DNMT* (*DNMT1*,* DNMT3av1*,* DNMT3av2*,* DNMT3bv1*,* DNMT3bv2*,* DNMT3bv3*), *MeCP2,* and *TET* (*TET2*,* TET3v1*,* TET3v2*) of adult tissues. Data were analyzed using GraphPad Prism software version 5.0.

## 3 RESULTS

### 3.1 Methylation of CpG genomic DNA is dynamic during the rivulus lifecycle

A significant difference in CpG global methylation of genomic DNA (*p *< 0.0001) was observed among tissues of adult rivulus (Table 1A). Gonad (87.22% ± 1.14, 79.55% ± 1.78; for males and hermaphrodites, respectively) and liver (80.93% ± 1.53, 81.67% ± 2.35; for males and hermaphrodites, respectively) were the most highly methylated organs while brain, gill, skin, and muscle exhibited significantly lower levels of methylation (Table 1B). However, when we compared CpG DNA methylation levels between males and hermaphrodites, we observed that only gonadal tissues exhibited significant sex differences, with male testes having higher levels of CpG DNA methylation than hermaphrodite ovotestes (Table 1B).

**Table 1:** CpG global DNA methylation levels in different organs of *Kryptolebias marmoratus* using the LUminometric Methylation Assay (LUMA)

(A)			
Source of Variation	*F* (DFn,DFd)	% of Total Variation	*p* Value
Interactions	*F* (5, 24) = 2.343	11.01	.0723
Sex	*F* (1, 24) = 4.755	4.467	.0392
Organs	*F* (5, 24) = 13.2	61.98	<.0001

(B)				
Tissue	DNA methylation Mean% (±*SEM*) (Males)	DNA methylation Mean% (±SEM) (Hermaphrodites)	Sidak's multiple comparisons test (between male and hermaphrodite organs)	Sidak's multiple comparisons test (between tissues)
Gonad	87.22 ± 1.14	79.55 ± 1.78	0.0058	A
Brain	78.22 ± 0.84	75.88 ± 3.68	0.8349	B
Liver	80.93 ± 1.53	81.67 ± 2.35	0.9995	C
Gills	76.05 ± 0.61	77.36 ± 0.57	>0.9999	B
Muscle	73.71 ± 1.28	73.73 ± 7.32	0.9307	B
Skin	73.71 ± 1.28	74.15 ± 1.96	>0.9999	B

Results of (A) two‐way ANOVA (*p* < 0.05) followed by (B) Sidak's post hoc test on adult tissues where different letters indicate tissues with significantly different mean DNA methylation.

**Figure 2:**
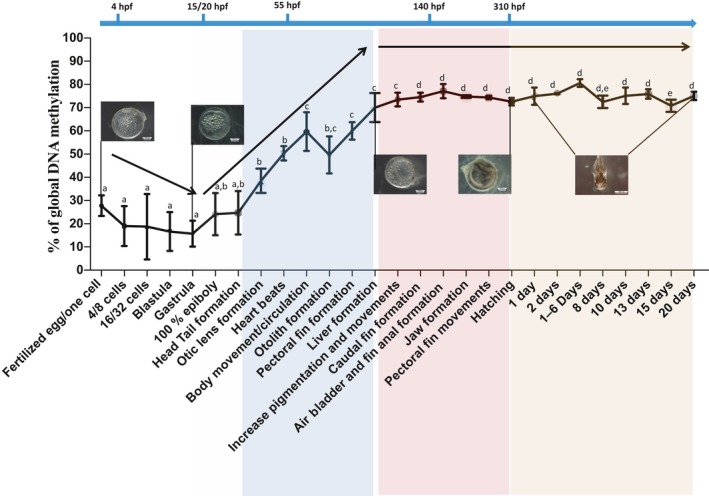
CpG DNA methylation levels during the embryonic development of *Kryptolebias marmoratus*. The results are given for a quantity of 600 ng of genomic DNA using the LUminometric Methylation Assay (LUMA) and the corresponding name stages of the Figure 2. The developmental stages are indicated under the *x*‐axis. *P* values are given for Tukey's post hoc test after one‐way ANOVA if *p* < 0.05 threshold. Means labeled with the same letter do not display statistically significant differences

Global methylation of CpG genomic DNA was not constant during early development (Figure 2). From a relatively low level of methylation in fertilized eggs (27.8% ± 4.4), rivulus DNA became significantly less methylated up to blastula/gastrula stages (17.0% ± 8.0 and 15.8% ± 5.6, respectively) in comparison with the otic lens formation stage (38.53% ± 5.0). CpG DNA methylation then increased until the liver formation stage (70.0% ± 6.0) and remained constant from the caudal fin formation stage (74.5% ± 1.9) until hatching (72.6% ± 1.6). This global CpG methylation level remained stable during the first 20 days of the juvenile stage (74.4% ± 0.8) except for a significant decrease at 15 dph (70.8% ± 2.6).

### 3.2 Rivulus DNMT enzyme family

Our approach led to the molecular characterization of six cDNAs encoding putative DNMT orthologues, namely *DNMT1*,* DNMT3av2*,* DNMT3bv1*,* DNMT3bv2,* and *DNMT3bv3*. These sequences had the DCM (DNMT1, DNMT3av1, DNMT3av2) or AdoMet‐MTase (DNMT3bv1, DNMT3bv2, DNMT3bv3) domains that putatively confer methyltransferase activity (Figure S2A). In addition, rivulus DNMT also displayed different conserved domains. Such domains (DNMT1‐RFD, DNMT3b‐Relat, PWWP) recognize and bind the correct residues (Figure S2A). The putative sequence was confirmed by PCR amplification of the full‐length molecule (Figure S2B).

Phylogenetic analysis identified two groups corresponding to rivulus DNMT and their cognate orthologues in a wide range of animal species (Figure S3). The first group corresponded to the maintenance DNMT1, which displayed maximum similarity with fish and more precisely *Xiphophorus sp*. The second group corresponded to *de novo* DNMT3a and DNMT3b. Interestingly, DNMT3bv1 was closer of DNMT3a. They also shared maximum of homology with the fish *Larimichthys crocea*, except for DNMT3av2, which was closer to *Danio rerio*.

**Figure 3:**
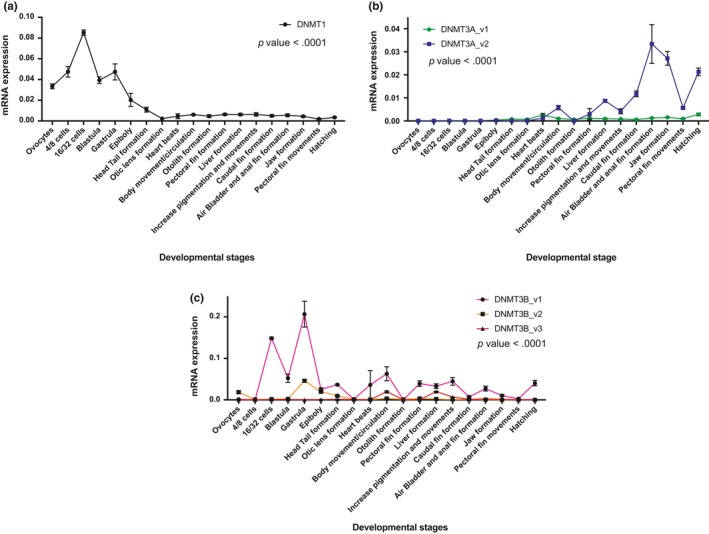
mRNA expression of Rivulus *DNMT* genes during early stages of *Kryptolebias marmoratus*. Expression levels relative to ß‐actin are given. The developmental stages are indicated under the *x*‐axis. *P* values are given for Tukey's post hoc test after one‐way ANOVA if *p* < .05 threshold. (a) *DNMT1*, (b) *DNMT3A_v1* and *DNMT3A_v2*, (c) *DNMT3B_v1*,*DNMT3B_v2* and *DNMT3B_v3*

**Figure 4:**
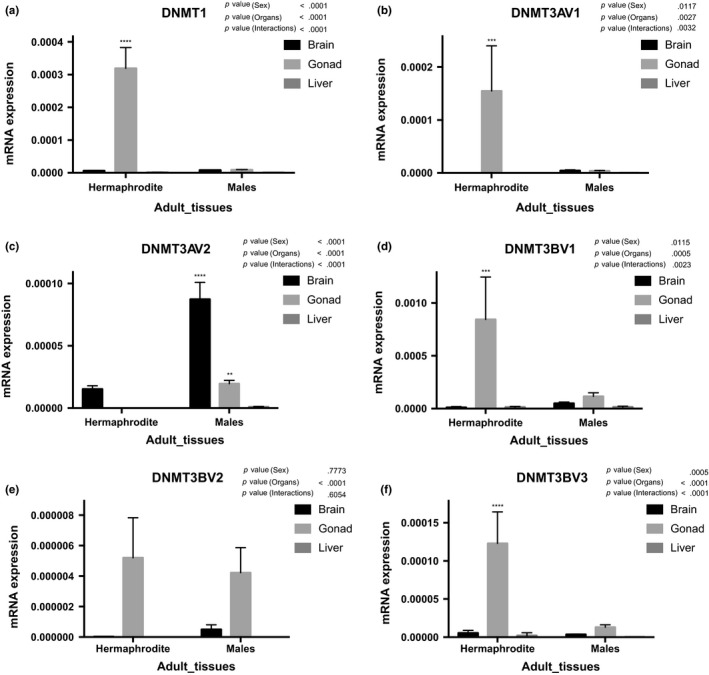
mRNA expression of Rivulus *DNMT* genes in adult tissues of *Kryptolebias marmoratus*. Expression levels relative to 18S‐RNA are given. The adult tissues are indicated under the *x*‐axis. *P* values are given for Sidak's post hoc test after two‐way ANOVA if *p* < .05 threshold. (a) *DNMT1*, (b) *DNMT3Av1*, (c) *DNMT3Av2*, (d) *DNMT3Bv1*, (e) *DNMT3Bv2,* and (f) *DNMT3Bv3*

Rivulus *DNMT*s displayed high expression levels with significant variation during early development (Figure 3). *DNMT1* mRNA levels increased from fertilized oocytes to the 16/32 cell stage and decreased at the blastula stage. Another peak was evident at the gastrula stage until otic lens formation. Transcript levels then remained stable until hatching (Figure 3a). In contrast, both *DNMT3a* mRNA variants increased irregularly from the heart beat stage to hatching, and *DNMT3av2* showed significantly higher expression levels than *DNMT3av1* (Figure 3b). *DNMT3b* mRNA variants were expressed irregularly during embryonic development. *DNMT3bv1* showed a very high level of expression from the 4/8‐cell stage to the gastrula stage (Figure 3c). In contrast, adult tissues exhibited low levels of all *DNMT* subtypes expression that were tissue‐specific (Figure 4). Furthermore, *DNMT1* (Figure 4a), *DNMT3av1* (Figure 4b), *DNMT3bv1* (Figure 4d), and *DNMT3bv3* (Figure 4f) displayed significantly higher mRNA expression in hermaphrodite ovotestes than male testes, while *DNMT3av2* (Figure 4c) showed significantly higher expression in male brain and testes than hermaphrodite brain and ovotestes, respectively.

### 3.3 Rivulus TET enzymes family

*In silico* analysis allowed molecular characterization of four cDNAs encoding putative TET orthologues, namely *TET1*,* TET2*,* TET3v1,* and *TET3v2*. These sequences had the TET_JBP domain, which is putatively able to confer 5‐hydroxymethyltransferase activity (Figure S4a). A zf‐CXXC motif was also present on rivulus TET1 (Figure S4a).

Phylogenetic analyses of rivulus TET proteins revealed two groups corresponding to the TET2 and TET1/TET3 in a wide range of animals (Figure S4B). The first group corresponded to the TET1/TET3 family. Interestingly, all rivulus TET proteins belonged to this group. Rivulus TET3 was very close to *Danio rerio* TET3. Furthermore, rivulus TET1 showed high homology with other killifish TET1. Finally, rivulus TET2 and also *Amazona aestiva* TET2 were more homologous with fish TET1/TET3 than TET2 in other vertebrates.

**Figure 5:**
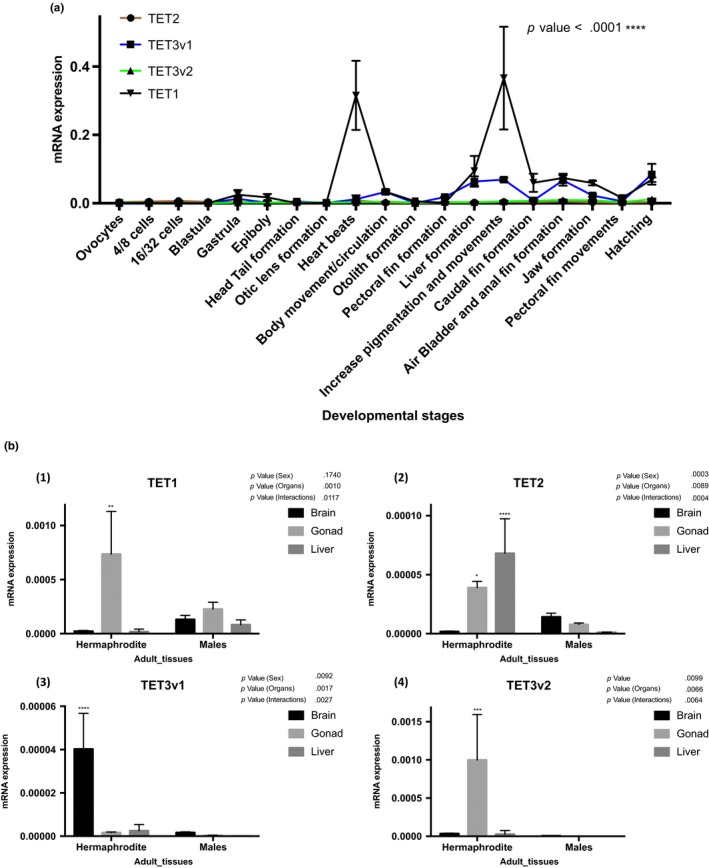
mRNA expression of Rivulus *TET* genes during early stages and adult tissues of *Kryptolebias marmoratus*. (a) Expression levels relative to ß‐actin are given. The developmental stages are indicated under the *x*‐axis. *P* values are given for Tukey's post hoc test after one‐way ANOVA if *p* < .05 threshold. (b) Expression levels relative to 18S‐RNA are given. The adult tissues are indicated under the *x*‐axis. P values are given for Sidak's post hoc test after two‐way ANOVA if *p* < .05 threshold. (1) *TET1*, (2) *TET2*, (3) *TET3v1*, (4) *TET3v2*

Rivulus *TET*s displayed irregular expression profiles with significant variation during early development. *TET1* was the most highly expressed followed by *TET3v1* mRNA in comparison with *TET2* and *TET3v2* mRNA, which exhibited the lowest levels (Figure 5a). *TET1* mRNA increased from the gastrula stage to hatching with two expression peaks in the heart beat stage and in the stage characterized by increased pigmentation and movement (Figure 5a). Similarly, *TET3v1* mRNA rose from the gastrula stage to hatching with peaks of expression in gastrula, body movements/circulation, liver formation, increase pigmentation and movements, air bladder and anal fin formation and hatching stages (Figure 5a). *TET* was expressed at low levels, and in a tissue‐specific fashion, in adults (Figure 5b). *TET1* (Figure 5B1), *TET2* (Figure 5B2), and *TET3v2* (Figure 5B4) mRNA displayed significantly higher expression in hermaphrodite ovotestes in comparison with male testes. *TET2* (Figure 5B4) exhibited higher expression in hermaphrodite livers and *TET3v1* (Figure 5B3) was significantly higher in hermaphrodite brains compared to the same tissues in males.

### 3.4 Rivulus MeCP2 MBD protein

A search in the genomic databases permitted discovery of one cDNA encoding a putative MeCP2 orthologue belonging to the Methyl‐CpG‐Binding Proteins (MBD) family. This sequence had two conserved domains (MeCP2‐MBD and DMP1) putatively responsible for transcriptional activation of osteoblast‐specific genes (Figure S5A).

Phylogenetic analysis revealed two groups of MeCP2 in a wide range of animals (Figure S5B). Interestingly, rivulus MeCP2 formed a specific cluster where only fish MeCP2 were present and shares more homology with the perciforme *Labrus bergylta* than the cyprinodontiform *Fundulus heteroclitus*.

**Figure 6:**
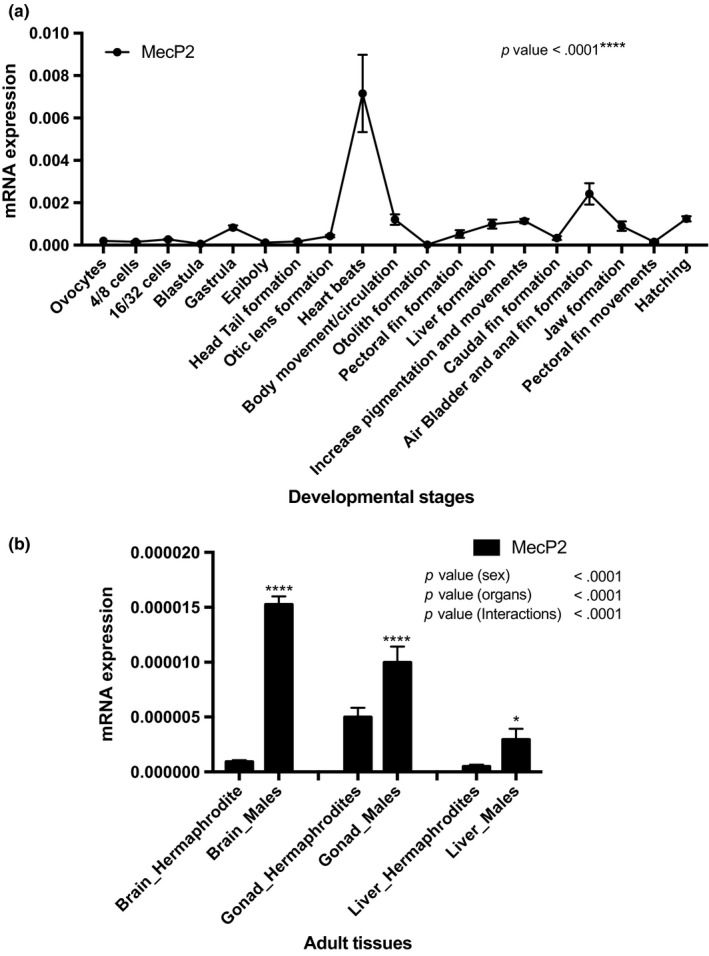
mRNA expression of Rivulus *MeCP2* genes during early stages and adult tissues of *Kryptolebias marmoratus*. (a) Expression levels relative to ß‐actin are given. The developmental stages are indicated under the *x*‐axis. *P* values are given for Tukey's post hoc test after one‐way ANOVA if *p* < .05 threshold. (b) Expression levels relative to 18S‐RNA are given. The adult tissues are indicated under the *x*‐axis. *P* values are given for Sidak's post hoc test after two‐way ANOVA if *p* < .05 threshold

*MeCP2* displayed high expression levels in embryos, with significant variation during early development and a peak at the heart beat stage (Figure 6a). In contrast, adult tissues expressed low levels of *MeCP2* expression (Figure 6b), but displayed significantly higher expression in male tissues with brain showing the highest, and liver the lowest. In hermaphrodites, *MeCP2* was significantly more expressed compared to other tissues.

## 4 DISCUSSION

Our study describes global CpG DNA methylation and mRNA expression of associated enzymes during development and adulthood within an isogenic lineage of mangrove rivulus, a species that allows the specific roles of epigenetic variation in producing phenotypic variability to be determined. Together, these results increase our understanding of epigenetic mechanisms that operate during fish embryonic development. Indeed, despite being well documented in plants and mammalian systems (Dean, Santos, & Reik, 2003; Feng et al., 2010; Shen et al., 2007; Swales & Spears, 2005), methylation studies during fish embryogenesis and gametogenesis (Labbé, Robles, & Paz, 2017) have focused mainly on *Danio rerio* (Martin et al., 1999; Mhanni & McGowan, 2004; Potok, Nix, Parnell, & Cairns, 2013) and, recently, on *Oryzias latipes* (Dasmahapatra & Khan, 2015; Walter, Li, Intano, Kazianis, & Walter, 2002).

In adult *K*. *marmoratus*, we reported that tissues were highly methylated at CpG sites with values between 73.7% in muscle and 87.2% in male testes, which is consistent with estimates obtained in other vertebrates; plants and invertebrates typically exhibit lower levels of DNA methylation (Feng et al., 2010; Head et al., 2014; Jabbari, Cacciò, Païs de Barros, Desgrès, & Bernardi, 1997; Rivière, 2014). Global CpG DNA methylation levels of adult somatic tissues were similar in males and hermaphrodites. On the contrary, and in line with *Danio rerio*, the male testis was hypermethylated compared to the hermaphrodite ovotestes (87.2% and 79.6%, respectively). Potok et al. (2013) demonstrated global CpG methylation of 95% in sperm and 75% in oocytes, whereas Laing et al. (2016) showed CpG methylation of 88% in sperm and 83% in oocytes. In the case of rivulus hermaphrodites, we could not differentiate male and female portions of the gonad, as the ovotestes included a mix of spermatogenic and ovarian tissue. Methylation level in the ovotestes is consequently an average of the two tissues and cannot be directly compared to a true female gonad. It is however reasonable to hypothesize that this methylation level is characteristic of ovarian tissue as only a small portion (most of the time <10/20%) (Soto, Leatherland, & Noakes, 1992) of the ovotestes contains spermatogenic tissue. The fact that the testis shows significantly higher DNA methylation indicates that in a self‐fertilizing hermaphrodite fish species, the differences between sperm and oocytes are comparable to what was reported in gonochoristic fish species like *D. rerio*.

**Figure 7:**
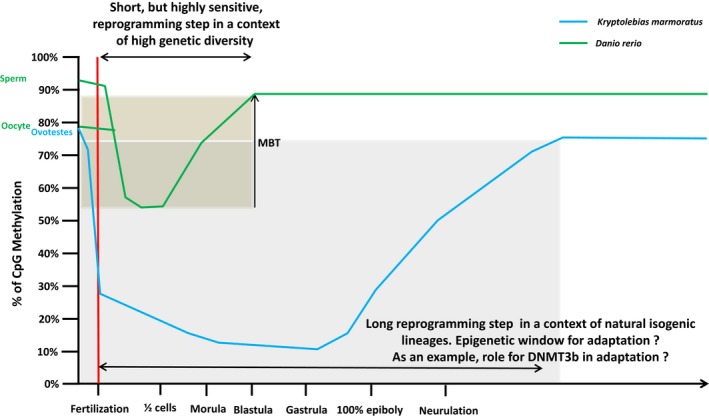
Dynamic of DNA methylation during early zebra fish and mangrove rivulus development. (MBT, Mid Blastula Transition)

During embryonic development, we reported that global CpG methylation of genomic DNA was dynamic. We found that the methylation profile of *K. marmoratus* is consistent with that of *Mus musculus* (Razin & Shemer, 1995) and *Danio rerio* (Mhanni & McGowan, 2004) genomes, for which DNA undergoes global demethylation promptly after fertilization and then becomes remethylated at specific loci and stages. These data point to global demethylation and subsequent remethylation as important in vertebrate development. However, unique to our knowledge, global CpG methylation of the rivulus genome increased from a remarkably low level (15.8% ± 5.6) at the end of the gastrula stage (15 hr postfertilization—hpf) before it stabilized around the liver formation stage (90 hpf), when the embryo is already well formed. Despite contradictory results (Macleod, Clark, & Bird, 1999), methylation of *D. rerio* increases from an intermediate level (~56%) in early‐blastula stage and is re‐established to adult levels at late gastrula stage, much earlier in embryonic development compared to rivulus (Figure 7) (Kamstra, Alestrm, et al., 2015; Kamstra, Løken, et al., 2015; Mhanni & McGowan, 2004). In *Oryzias latipes*, despite the presence of DNMT enzymes with a distinct transcriptional pattern during early development (Dasmahapatra & Khan, 2015) and a time of development (6 days) that falls between that of *D*. *rerio* and *K*. *marmoratus* (Iwamatsu, 2004), DNA methylation in embryos seems to be characterized by a lack of global methylation pattern erasure during reprogramming (Walter et al., 2002). These data suggest, from a comparative standpoint at similar embryonic stages and despite different times of development, that fish embryogenesis is regulated in a different manner among species. Also, further studies should investigate in *K. marmoratus* whether the methylome is inherited from the sperm and not the oocyte, as demonstrated in *D. rerio* (Jiang et al., 2013). In this latter species, the oocyte‐specific methylation profile is indeed gradually discarded and reset to the sperm methylome pattern (Hackett & Surani, 2013). If this mechanism happens in *K. marmoratus* together with a deep, late, and long reprogramming event, thus, elucidation of the epigenetic mark transmission, and of the potential impact of embryonic demethylation on inbreeding depression limitation (Vergeer, Wagemaker, & Ouborg, 2012), might provide clues about the evolutionary pathways leading to this mix‐mating system and differences in selfing rates reported among natural populations. Stability of this mixed mating system is indeed possibly linked to the magnitude of inbreeding depression that is affected by environmental conditions (Cheptou & Donohue, 2013), and self‐fertilization increases homozygosity with, in consequences, the fitness reduction of individuals (Venney, Johansson, & Heath, 2016). However, in *K*. *marmoratus*, selfing maintain locally well‐adapted genotype (Avise & Tatarenkov, 2012) and the study of this unique reprogramming pattern might be a key to explore. In a recent study, Vergeer et al. (2012) have indeed demonstrated that inbreeding depression was nearly completely removed after chemical demethylation of *Scabiosa columbaria* seedlings. Furthermore, specific regulation of DNA methylation during reprogramming appears to be crucial in fish embryogenesis with developmental abnormalities when DNA methylation is inhibited during the remethylation phase in blastula stage (Dasmahapatra & Khan, 2015; Martin et al., 1999). Together with a high level of DNA CpG demethylation, the reprogramming period in rivulus is long compared to other studied vertebrate species (mammals and fish) (Haaf, 2006; Mhanni & McGowan, 2004; Walter et al., 2002) and is achieved at a late embryonic stage. This pattern might partly be explained by the longer developmental time of rivulus (310 hpf) compared to *D*.* rerio* (48–72 hpf). However, mammals exhibit an early remethylation time (8 to 16 cell stage for bovine embryos, begins only in the blastocyst in mouse) despite a long embryonic development period (reviewed in Haaf, 2006). As the remethylation event is accepted as a critical step during embryogenesis (Martin et al., 1999; Mcgee, Cooper, Stapleton, & Volz, 2012), the long apparent reprogramming period observed during rivulus development may represent a relatively prolonged critical window especially sensitive to environmental cues. Further studies should investigate the evolutionary significance for rivulus to present such a prolonged critical window. Because rivulus live in highly variable mangrove environments, this long DNA methylation reprogramming period could permit environmental signals to be translated and assimilated at the epigenetic level during embryogenesis and could consequently increase phenotypic diversity. This suggested integration of environmental cues in the epigenome might facilitate the expression of new phenotypes in lineages characterized by a low level of genetic diversity (Figure 7), in accordance with the general‐purpose genotype (GPG) model. This model proposes that evolutionary success of isogenic lineages could be possible via generalist individuals selected for their plastic phenotypes utilizing wide ecological niches (Massicotte & Angers, 2012).

The importance of the reprogramming event (Dasmahapatra & Khan, 2015; Martin et al., 1999) and the dynamic pattern of global CpG DNA methylation in rivulus suggest a precise regulation in time and space of epigenetic marks by the six putative DNMT proteins characterized in *K*. *marmoratus*. The presence of the catalytic conserved domains (DCM, Adomet‐MTase) suggests that rivulus DNMTs might be active proteins (Song, Teplova, Ishibe‐Murakami, & Patel, 2012; Turek‐Plewa & Jagodzinski, 2005), whereas additional evolutionary conserved domains (DNMT1‐RFD, BAH, PWWP) might confer enzymatic specificities between orthologues. Phylogenetic analysis showed their divergent evolution (Dabe, Sanford, Kohn, Bobkova, & Moroz, 2015) indicating a role in the maintenance of DNA methylation patterns for DNMT1, and one as methyltransferases responsible for *de novo* establishment of DNA methylation profiles for DNMT3a/b, which might be conserved. Consistent with previous observations (Kim et al., 2016), our results suggest that DNMT1 is a monophyletic group with a conserved structure among metazoan (Dabe et al., 2015). On the other hand, rivulus DNMT3 family formed three subgroups (DNMT3bv1, DNMT3av1/v2, and DNMT3bv2/v3). Interestingly, DNM3bv1 is closer to DNMT3a than to the other DNMT3b variants, which might indicate a common biological function, whereas the DNMT3bv2/v3 may have a distinct function. DNMT3bv1 and DNMT3a share, indeed, a DNA‐binding domain and have a molecular size similar to what has been observed in their mammalian orthologues and cluster into the two main clades known in mammals as DNMT3a and DNMT3b (Turek‐Plewa & Jagodzinski, 2005). In contrast, DNMT3bv2/3 could be related to DNMT3ba, a fish‐specific DNMT3 gene lineage subclade (Campos et al., 2012; Dasmahapatra & Khan, 2015; Firmino et al., 2017; Shimoda, Yamakoshi, Miyake, & Takeda, 2005; Takayama, Shimoda, Takanaga, Hozumi, & Kikuchi, 2014) that result from fish‐specific duplication. Moreover, as in zebrafish, frogs, and chickens, extensive searches against the putative *dnmt3l* in *K*. *marmoratus* have failed suggesting that this gene likely appear during the mammalian evolution and might be associated with the evolution of genomic imprinting (Campos et al., 2012). In adults, *DNMT* expression levels were weak outside of the gonad. This mRNA expression level, higher in ovotestes except for *DNMT3av2*, indicates a potential role of rivulus DNMT in oogenesis even if we cannot exclude a putative function in spermatogenesis as in mammals (Uysal, Akkoyunlu, & Ozturk, 2016). This hypothesis matches the situation in *D. rerio* where DNMT1 is found with high abundance in the unfertilized egg (Fang et al., 2013). High expression levels of rivulus *DNMT1* mRNA in early stages, from fertilized oocytes to epiboly, might show a maternal origin and is reminiscent of the situation in other fish species (Dasmahapatra & Khan, 2015; Firmino et al., 2017). Up‐regulation of *DNMT3av2* from the otic lens formation stage till hatching is similar to other fish (Dasmahapatra & Khan, 2015; Fang et al., 2013; Firmino et al., 2017) where DNMT3a is implicated in lens formation (Seritrakul & Gross, 2014), neurogenesis (Firmino et al., 2017), and organogenesis (Takayama et al., 2014). Based on these results, as in zebrafish (Campos et al., 2012), we suggest that DNMT3a and DNMT3b play different roles, and we hypothesize that some level of erasure and repatterning of *de novo* CpG DNA methylation might be taking place early in embryogenesis (Figure 7) with *DNMT3bv1*, and later during the remethylation phase with DNMT3av2.

In addition to DNA methylation, DNA 5‐hydroxymethylation (5hmC), catalyzed by TET enzymes, was recently characterized in vertebrate genomes (Tahiliani et al., 2009). Even if the level of 5 hmC is still unknown in mangrove rivulus, *in silico* analyses revealed four genes putatively coding for TET enzymes. Rivulus TET proteins clustered within two distinct groups with TET2 on the one hand and TET1/TET3 on the other hand. Interestingly, rivulus TET2 cluster, with other teleosteans, within the TET1/TET3 group exclusively composed of teleost fishes and do not share high homology with other vertebrate TETs, the closest being birds. Furthermore, the rivulus TET family is characterized by a lack of CXXC motif in TET3 but not in TET1, a distinct feature of the TET1/3 family in mammals (Tan & Shi, 2012). We argue, based on the data, that TET may have an additional and/or different biological role or activity in fishes. Indeed, signs of functional divergence and adaptive evolution of the TET family have already been observed among diverse mammalian lineages (Akahori, Guindon, Yoshizaki, & Muto, 2015; Jafarpour et al., 2017). Finally, the presence of a catalytic domain suggests that rivulus TET proteins might be active. Adult rivulus exhibit only weak *TET* expression in comparison to late embryos. However, the significantly higher level of *TET3v1* transcript in hermaphrodite brains as of *TET1*/*TET2* in male brains, even if the values are not significantly different, suggest a conserved role for rivulus TET proteins in neurogenesis, with a potential specific function of TET3v1 in hermaphrodite brains, as in *M*. *musculus*,* Xenopus*. sp and *D*. *rerio* (Diotel et al., 2017; Wang, Tang, He, & Jin, 2016). In these species, *TET2* and *TET3* mRNA increased from Neural Progenitor Cells (NPC) to neurons (Diotel et al., 2017; Wang et al., 2016) and *M*. *musculus* lacking TET1 showed impaired hippocampal neurogenesis, and poor learning and memory (Wang et al., 2016). High expression levels of *TET1*/*TET2*/*TET3v2* in ovotestes and *TET1* in testes indicate a putative role in oocytes and spermatozoa maturation as in humans, where dynamic expression of *TET* is associated with male fertility (Ni et al., 2016). Furthermore, female *M*. *musculus* depleted of TET3 in the germ line show severely reduced fecundity (Gu et al., 2011). Finally, *TET* expression patterns during embryonic development indicate a putative biological function in organogenesis. As an example, in comparison with *TET2*/*TET3*,* TET1* showed a high expression in the heart beat stage, characterized by development of the nervous system (Mourabit et al., 2011), and in the liver formation stage. These data are in accordance with *D*. *rerio* for which these proteins are essential to embryonic development (Gu et al., 2011; Li et al., 2015) with a level of *TET1* higher than *TET2*/*TET3* and an increase of *TET* transcript from 24h to 48h postfecundation during organogenesis (Almeida et al., 2012; Ge et al., 2014). However, between the two TET3 variants, the high transcript expression of rivulus *TET3v1* compared to *TET3v2* may indicate that the isoform 1 regulates the development in normal conditions whereas the isoform 2 might rescue it, even if two specific functions are possible.

Finally, we characterized one gene coding for MeCP2, a member of the MBD family, in rivulus. However, new analysis might reveal other MBD members in this species as five proteins (MBD1, MBD2, MBD3, MBD4, and MeCP2) have been described in vertebrates (Ballestar & Wolffe, 2001; Roloff, Ropers, & Nuber, 2003; Wade, 2001). While the role of DNA methylation and DNMT enzymes has been well characterized in mammalian development (Dean et al., 2003; Swales & Spears, 2005), the mechanisms of MBD, generally thought to govern normal embryogenesis, are still largely unknown (Bogdanovi & Veenstra, 2009). Our analyses show that rivulus MeCP2 clusters with teleost species, which might argue for specific functions within teleosteans. In adults, a significantly higher level of *MeCP2* transcript was found in male gonads and brains, indicating a putative role in spermatogenesis and neurogenesis. In mammals, indeed, *M*. *musculus* that were knockout for the *MeCP2* gene exhibit a phenotype similar to Rett syndrome, a human neurodevelopmental disorder associated with *MeCP2* mutations (Carney et al., 2003; Collins et al., 2004; Vieira Pedro et al., 2015). Interestingly, MeCP2‐null *D*. *rerio* are viable and fertile but exhibit a reduction in activity and a decrease in anxiety‐like behavior (Pietri et al., 2013). The dynamic pattern of *MeCP2* later in development, particularly in the heart beat stage, suggests also involvement neurogenesis, which is in line with *D*. *rerio* where MeCP2 regulates neural cell differentiation (Gao et al., 2015) and is required for proper axonal elongation of motor neurons and synapse formation (Nozawa et al., 2017). Finally, we hypothesize that the high level of rivulus *TET1* and *MeCP2* in the heart beat stage signifies a putative interaction already observed in the turtle *Trachemys scripta elegans* where MeCP2 regulates the DNA methylation status indirectly through association with TET1 protein (Zheng, Ambigapathy, & Keifer, 2017). However, further works are required to understand links between MeCP2, TET, and DNA methylation (Fasolino & Zhou, 2017) and to resolve their functional consequences on gene expression.

## 5 CONCLUDING COMMENTS

In this study, we presented an epigenetic basis for DNA methylation in *Kryptolebias marmoratus*, a species unique among vertebrates because of its ability to naturally produce isogenic lineages through self‐fertilization. This new model species allows researchers to specifically examine the epigenetic contributions to phenotypic variation and to investigate more deeply the relationships between environment, epigenome, and phenotype. We characterized a specific pattern of CpG DNA methylation reprogramming during embryogenesis, which is later, deeper, and longer than reported in other vertebrates, associated with dynamic mRNA expression of DNA methylation/demethylation enzyme machinery that interrogate on the precise role of each isoforms. Our results raise questions about the evolutionary significance of this reprogramming event, hypothesized as a putative critical window mediating phenotypic plasticity and evolutionary adaptation to highly variable environments, and about the inheritance of epigenetic marks in a self‐fertilized species. Also, because wild populations of rivulus consist of many heterozygous and homozygous lineages, our work, which establishes a basis to study DNA methylation in *K*. *marmoratus*, raises question about whether populations vary in their epigenetic profiles, and on interactions between epigenetic modifications and selfing rates in relation with environmental challenges, mate availability and inbreeding depression. Furthermore, phenotypic plasticity early in development together with large eggs (easily identifiable embryonic stages) and the important capacities for physiological adaptation to challenging mangrove environments might make rivulus an important tool for advancing our understanding of the epigenetic machinery. Already a valuable model organism in ecotoxicology, behavior, physiology, and evolutionary biology, we propose *K*. *marmoratus* as a valuable new vertebrate model to explore the role of DNA methylation within vertebrate development and reproduction and to investigate ecological and evolutionary epigenetics.

## CONFLICT OF INTEREST

The authors declare that they have no conflict of interest.

## AUTHOR CONTRIBUTIONS

Conceived and designed the experiments: Fellous A, Silvestre F. Performed the experiments: Fellous A, Labed‐Veydert T, Voisin AS, Locrel M. Analyzed the data: Fellous A, Earley R, Silvestre F. Wrote the manuscript: Fellous A, Earley R, Silvestre F.
