# Supplementary material for: DNA methylation in adults and during development of the self‐fertilizing mangrove rivulus, Kryptolebias marmoratus
Source: Ecol Evol. 2018 May 15;8(12):6016–33. doi: 10.1002/ece3.4141 (PMC6024129; doi:10.1002/ece3.4141)
Supplement: Supplementary file 1 [file ECE3-8-6016-s001.pptx]

## Slide 1
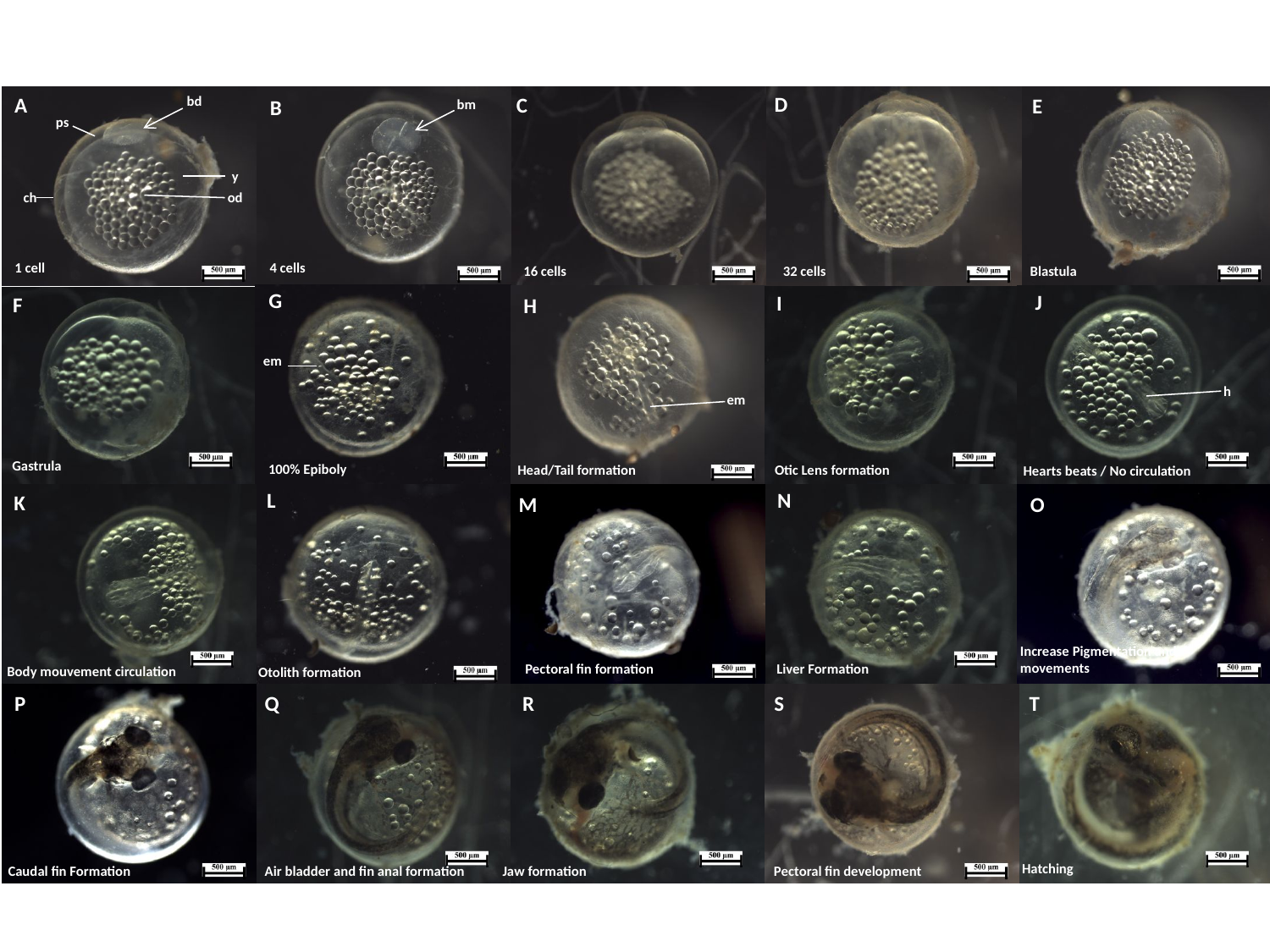

D
C
A
bd
E
B
bm
ps
y
ch
od
1 cell
4 cells
16 cells
32 cells
Blastula
G
J
I
F
H
em
h
em
Gastrula
100% Epiboly
Head/Tail formation
Otic Lens formation
Hearts beats / No circulation
N
L
K
O
M
Increase Pigmentation and movements
Pectoral fin formation
Liver Formation
Body mouvement circulation
Otolith formation
T
S
R
P
Q
Hatching
Pectoral fin development
Caudal fin Formation
Air bladder and fin anal formation
Jaw formation
