# Supplementary material for: DNA methylation in adults and during development of the self‐fertilizing mangrove rivulus, Kryptolebias marmoratus
Source: Ecol Evol. 2018 May 15;8(12):6016–33. doi: 10.1002/ece3.4141 (PMC6024129; doi:10.1002/ece3.4141)
Supplement: Supplementary file 2 [file ECE3-8-6016-s002.pptx]

## Slide 1
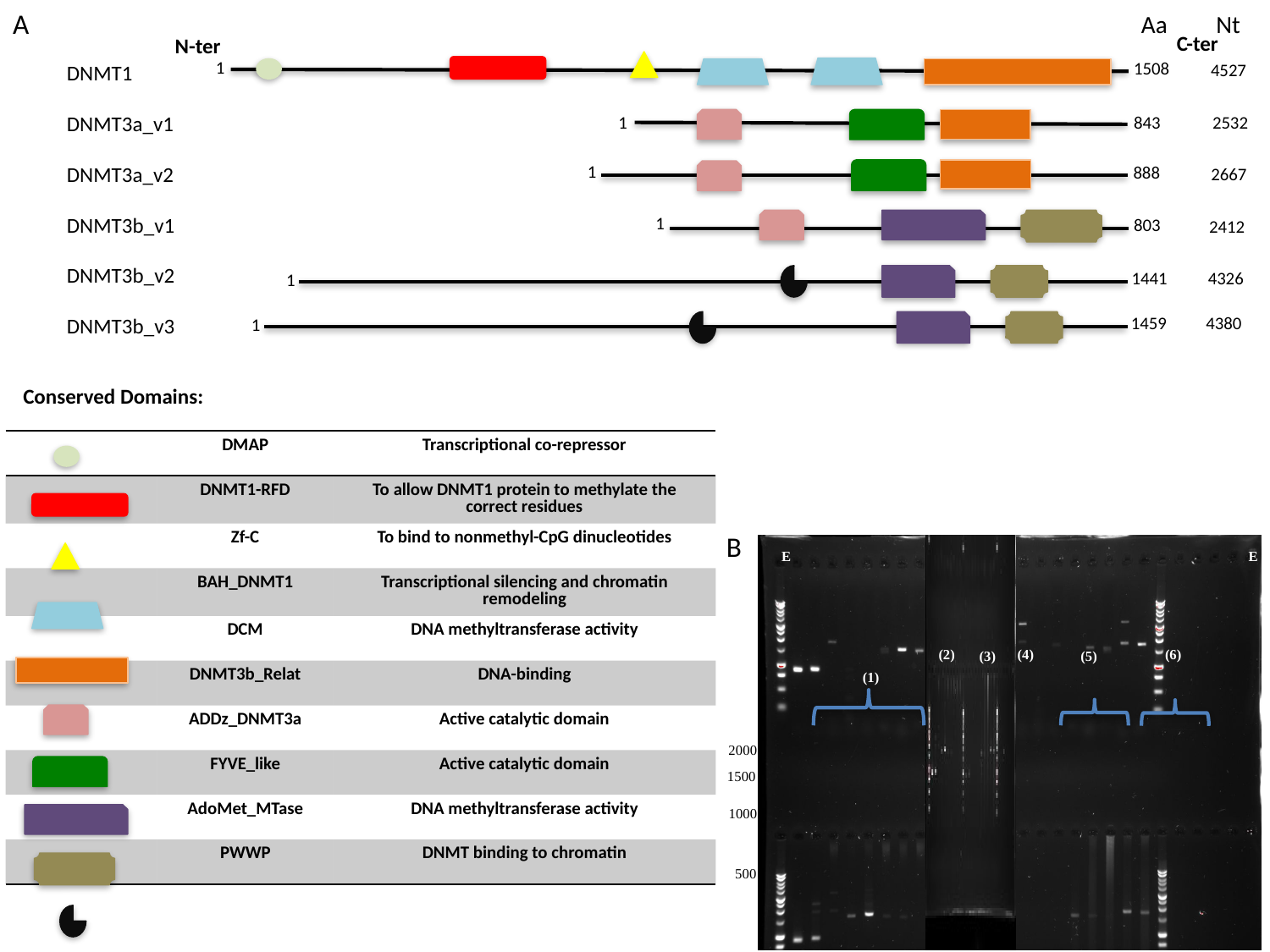

A
DNMT1
DNMT3a_v1
DNMT3a_v2
DNMT3b_v1
DNMT3b_v2
DNMT3b_v3
Aa
Nt
C-ter
N-ter
1
1508
4527
1
2532
843
1
888
2667
1
803
2412
1441
4326
1
1459
4380
1
Conserved Domains:
| | DMAP | Transcriptional co-repressor |
| --- | --- | --- |
| | DNMT1-RFD | To allow DNMT1 protein to methylate the correct residues |
| | Zf-C | To bind to nonmethyl-CpG dinucleotides |
| | BAH\_DNMT1 | Transcriptional silencing and chromatin remodeling |
| | DCM | DNA methyltransferase activity |
| | DNMT3b\_Relat | DNA-binding |
| | ADDz\_DNMT3a | Active catalytic domain |
| | FYVE\_like | Active catalytic domain |
| | AdoMet\_MTase | DNA methyltransferase activity |
| | PWWP | DNMT binding to chromatin |
B
E
E
(1)
2000
1500
1000
500
(2)
(4)
(6)
(3)
(5)
