# Supplementary material for: DNA methylation in adults and during development of the self‐fertilizing mangrove rivulus, Kryptolebias marmoratus
Source: Ecol Evol. 2018 May 15;8(12):6016–33. doi: 10.1002/ece3.4141 (PMC6024129; doi:10.1002/ece3.4141)
Supplement: Supplementary file 3 [file ECE3-8-6016-s003.pptx]

## Slide 1
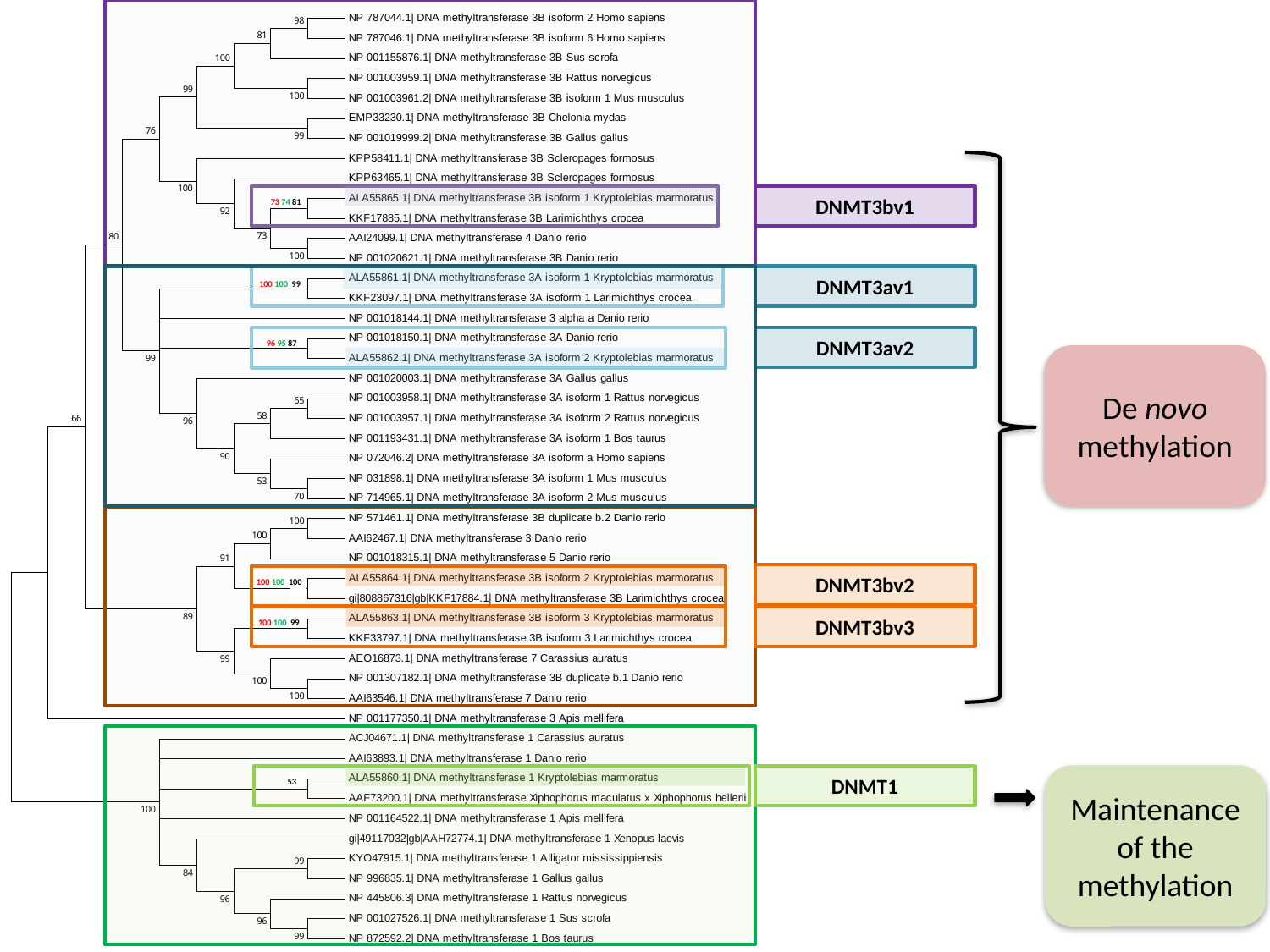

DNMT3bv1
73 74 81
DNMT3av1
100 100 99
DNMT3av2
96 95 87
De novo methylation
DNMT3bv2
100 100 100
DNMT3bv3
100 100 99
DNMT1
Maintenance of the methylation
53
