# Supplementary material for: DNA methylation in adults and during development of the self‐fertilizing mangrove rivulus, Kryptolebias marmoratus
Source: Ecol Evol. 2018 May 15;8(12):6016–33. doi: 10.1002/ece3.4141 (PMC6024129; doi:10.1002/ece3.4141)
Supplement: Supplementary file 4 [file ECE3-8-6016-s004.pptx]

## Slide 1
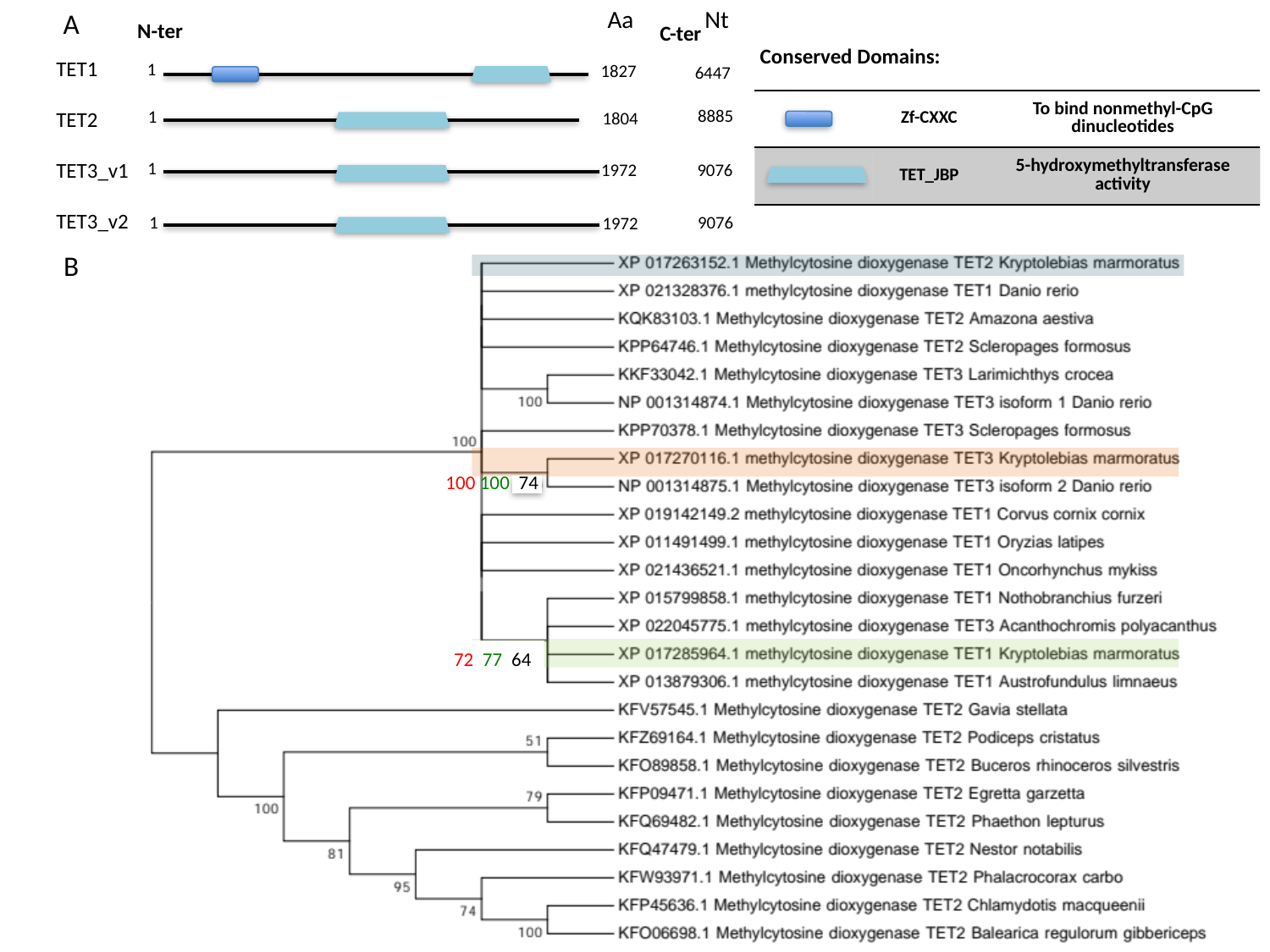

Aa
Nt
A
N-ter
C-ter
Conserved Domains:
TET1
TET2
TET3_v1
TET3_v2
1
1827
6447
| | Zf-CXXC | To bind nonmethyl-CpG dinucleotides |
| --- | --- | --- |
| | TET\_JBP | 5-hydroxymethyltransferase activity |
8885
1
1804
1
1972
9076
1
9076
1972
B
100 100 74
72 77 64
