# Supplementary material for: DNA methylation in adults and during development of the self‐fertilizing mangrove rivulus, Kryptolebias marmoratus
Source: Ecol Evol. 2018 May 15;8(12):6016–33. doi: 10.1002/ece3.4141 (PMC6024129; doi:10.1002/ece3.4141)
Supplement: Supplementary file 5 [file ECE3-8-6016-s005.pptx]

## Slide 1
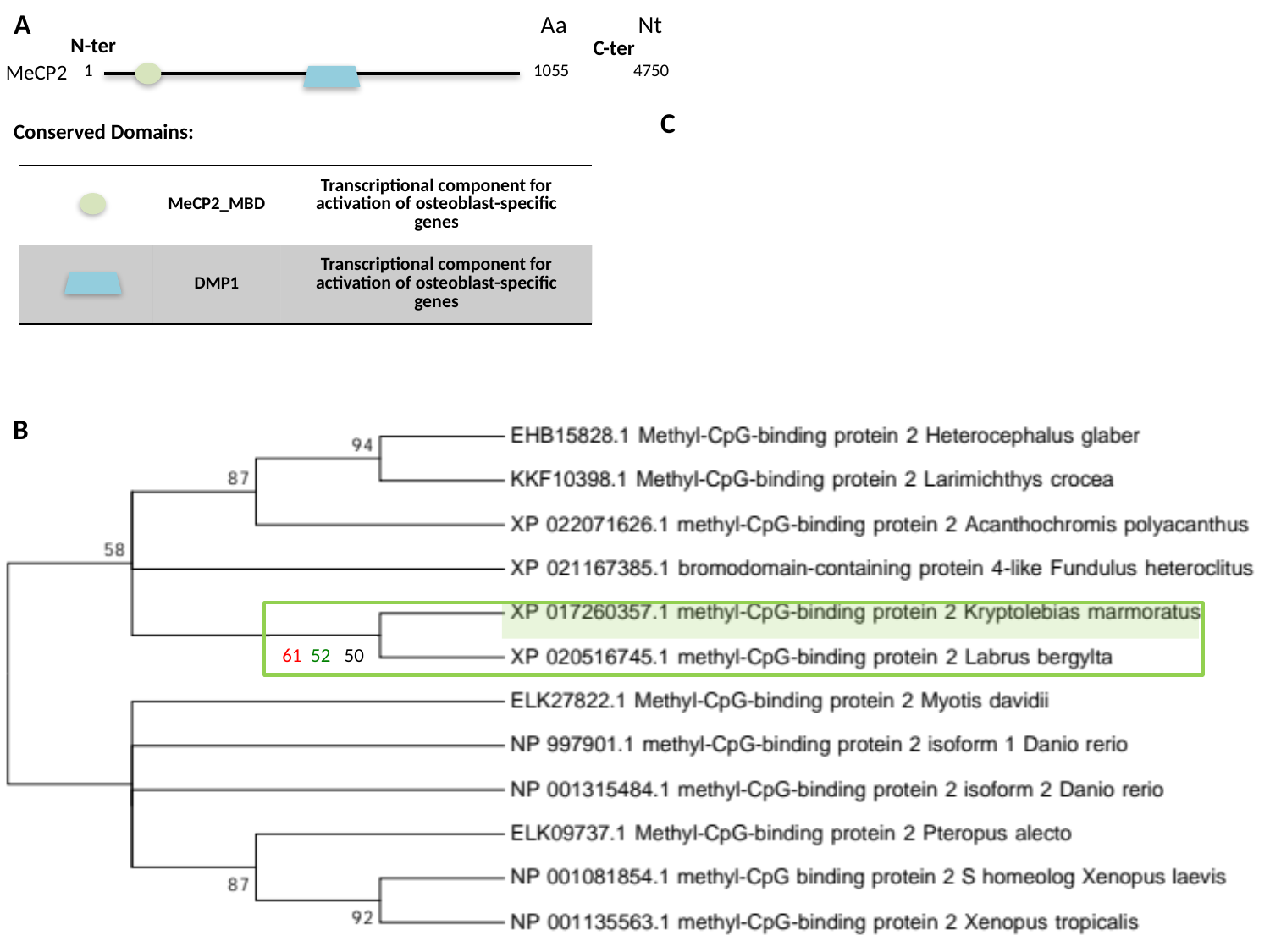

A
Aa
Nt
N-ter
C-ter
MeCP2
1
1055
4750
C
Conserved Domains:
| | MeCP2\_MBD | Transcriptional component for activation of osteoblast-specific genes |
| --- | --- | --- |
| | DMP1 | Transcriptional component for activation of osteoblast-specific genes |
B
61 52 50
