# Supplementary material for: DNA methylation in adults and during development of the self‐fertilizing mangrove rivulus, Kryptolebias marmoratus
Source: Ecol Evol. 2018 May 15;8(12):6016–33. doi: 10.1002/ece3.4141 (PMC6024129; doi:10.1002/ece3.4141)
Supplement: Supplementary file 6 [file ECE3-8-6016-s006.docx]

Supplementary Data Table

**Figure 1. Early embryonic development in *Kryptolebias marmoratus* showing stage transition from the first cell to hatching.** Images until the gastrula stages are in lateral view and from 100% epiboly to hatching are in head views of the embryos. Stage names are indicated at the bottom left. (Bd: blastodisc; bm: blastomeres; ch: chorion; dl: dorsal lip; em: embryo; od: oil droplet; ps: perivitelline space; y: yolk. Scale bar = 500 μm.)

**Figure 2**. **Predicted structure of the DNMT proteins in *Kryptolebias marmoratus*.** (A) Diagrams showing the conserved domain organization of the putative proteins encoded by the rivulus *DNMT* sequences. Gene names, proteins primary sequence length and cDNAs nucleotide sequence length are indicated. DNMT1-RFD (Cytosine Specific DNA Methyltransferase Replication Foci Domain), Zf-C (C-Type Zinc Finger), BAH_DNMT1 (Bromo-Adjacent Homology DNMT1 Domain), DCM (DNA Methyltransferase Domain), DNMT3b_Relat (S-Adenosyl-L-Methionine DNMT3b Domain), ADD (ATRX-DNMT3-DNMT3L (related to the plant homology (PHD)-like domain of regulator ATRX)), FYVE_Like (FYVE Zinc Finger Domain), AdoMet_MTase (S-Adenosyl methionine domain), PWWP (Pro-Trp-Trp-Pro). (B) cDNA amplification. (1) DNMT1 (3 fragments corresponding to the full-length amplification), (2) DNMT3A_v1, (3) DNMT3A_v2, (4) DNMT3B_v1, (5) DNMT3B_v2 (2 fragments corresponding to the full-length amplification), (6) DNMT3B_v3 (2 fragments corresponding to the full-length amplification).

**Figure 3**. **Phylogenetic analysis of the rivulus DNMT proteins.** Phylogenetic tree showing the rivulus DNMT family and their orthologues in the animal kingdom. Protein that cluster together share a common frame on the right. Numbers in the branches represent the bootstrap values (%) from 500 replicates obtained by the maximum likelihood method, the Neighbor Joining method (Green) and the Minimum Evolution method (Red).

**Figure 4**. **Predicted structure and phylogenetic analysis of the rivulus TET proteins.** (A) Diagrams showing the conserved domain organization of the putative proteins encoded by the rivulus *TET* sequences. Gene names, proteins primary sequence length and cDNAs nucleotide sequence length are indicated. TET_JBP (Ten-Eleven Translocation J-Binding Protein). (B) Phylogenetic tree showing the rivulus TET family and their orthologues in the animal kingdom. Protein that cluster together share a common frame on the right. Numbers in the branches represent the bootstrap values (%) from 500 replicates obtained by the maximum likelihood method, the Neighbor Joining method (Green) and the Minimum Evolution method (Red).

**Figure 5**. **Predicted structure and phylogenetic analysis of the rivulus MeCP2 proteins.** (A) Diagrams showing the conserved domain organization of the putative proteins encoded by the rivulus *MeCP2* sequences. Gene names, proteins primary sequence length and cDNAs nucleotide sequence length are indicated. MeCP2-MBD (Methyl-CpG binding protein 2 methyl-CpG binding **domain),** DMP1 (Dentin matrix acidic phosphoprotein). (B) Phylogenetic tree showing the rivulus MeCP2 family and their orthologues in the animal kingdom. Protein that cluster together share a common frame on the right. Numbers in the branches represent the bootstrap values (%) from 500 replicates obtained by the maximum likelihood method, the Neighbor Joining method (Green) and the Minimum Evolution method (Red).

**Table 1. GenBank accession number of the *DNMT*, *TET*, *MeCP2* and of the reference genes used**

| Gene name | GenBank accession number |
| --- | --- |
| *DNMT1* | ALA55860.1 |
| *DNMT3av1* | ALA55861.1 |
| *DNMT3av2* | ALA55862.1 |
| *DNMT3bv1* | ALA55865.1 |
| *DNMT3bv2* | ALA55864.1 |
| *DNMT3bv3* | ALA55863.1 |
| *TET1* | KT362994.1 |
| *TET2* | XP_017263152.1 |
| *TET3v1* | XM_017414627.1 |
| *TET3v2* | XM_017414628.1 |
| *MeCP2* | XP_017260357.1 |
| *ß-actin* | XM_017416676.1 |
| *18S RNA* | FJ438821 |

Table 2. **Primers used in PCR to obtain the DNMT mRNA sequence of the mangrove rivulus *Kryptolebias marmoratus*.**

| Primer | Sequence (5’- 3’) |
| --- | --- |
| DNMT1_L | GTA AAG AAC TTC ATC TGG AAA ATG GCT CTC |
| DNMT1_R | AGA TGT AGT CGG CAA AAG ATG TTG TGA AAC |
| DNMT1_L_1 | CTC GTA TGA GGA CCT TCT CAA CAA GATT |
| DNMT1_R_1 | GTA CGT GAT GTT CAC TTT TCC TTG GAC ATA |
| DNMT1_L_2 | CCG AGT ATT ACA GGA AGT CAT CAG ACT ACA |
| DNMT1_R_2 | CTT CAT CCT CTC CAG GAC ACA CTT CTT TAG |
| DNMT3A_v1_L | AAT CTA TGA AGT GTT ACA GGT GGC TAG CAG |
| DNMT3A_v1_R | GTA GTA ATC TTT GAG GAG TGG TGC AAA CAG GT |
| DNMT3A_v2_L | TTC TCT GTA GTT TGT GTG GAG AAA CTG ATG |
| DNMT3A_v2_R | CTA GTA GCA GGC AAA GTA CTC CTT GAG C |
| DNMT3B_v1_L | TGG GAC TCT TAT TGT GAG AAC AAG TCT GTA |
| DNMT3B_v1_R | GTA AAA CTC AAA GAA CAA CCT CCC TGT ACC |
| DNMT3B_v2_L_1 | AAC ACA CCT TTT GTG CTG TAC CTG TAT GTC |
| DNMT3B_v2_R_1 | TGT CTG TGT AGT CTG TGT TGT CTA CAC TCC |
| DNMT3B_v2_L_2 | CAG CAG GCA GAG TAT ATC TTC ATT GTGAC |
| DNMT3B_v2_R_2 | CTC ACA AAC CTC AGA GGC AAC ATA TTTATC |
| DNMT3B_v3_L_1 | AAG TGA GGA GGA CTG TAA GCA CAA CTT TAG |
| DNMT3B_v3_R_1 | AGC TGA TAC TCA TGG ATC TCT CTT TTG AGT |
| DNMT3B_v3_L_2 | GAC TCC CAG GCT CAC ACT ATA GAA ATT AAA |
| DNMT3B_v3_R_2 | TGA CTT GTG CAG CAA GAG ATG AAG TGA TAG |

Table 3. **Primers used in RT-qPCR to measure the mRNA expression of DNMT, MeCP2 and TET enzymes during the embryonic development and in adult tissues of the mangrove rivulus *Kryptolebias marmoratus*.** **Efficiency was calculated on a pool of cDNA corresponding to all the embryonnic stages and adult tissues used**

| Primer | Sequence (5’- 3’) | Efficiency |
| --- | --- | --- |
| DNMT1_L | GCT ATC CGT CAC CCG ACT AA | 90 % |
| DNMT1_R | TCA GCA AAG AAG GCA TCA AA | 90 % |
| DNMT3A_v1_L | ATG TTG GAC ACG TCG GTG TA | 92 % |
| DNMT3A_v1_R | GAC ACG CTC CAA CTC TGT GA | 92 % |
| DNMT3A_v2_L | GCT GTC AGA GGC CTG TTC AT | 101 % |
| DNMT3A_v2_R | TCG ACG CTA AGG AGG TGT CT | 101 % |
| DNMT3B_v1_L | GGA GGT CGA GAA GTT CGT | 90 % |
| DNMT3B_v1_R | CTC CGG GGT CAA TCA GGA TG | 90 % |
| DNMT3B_v2_L | CTG GCA GGA CTT GTT GGA AT | 94 % |
| DNMT3B_v2_R | TGG GAG AGG AAC AGG AAA TG | 94 % |
| DNMT3B_v3_L | TTC AAA GGT TTG CAC ACA GC | 95 % |
| DNMT3B_v3_R | CGT TGT GGT TGG TTT TTG TG | 95 % |
| TET1_L | GGG ACGTCT CTA AAG CCA CC | 108 % |
| TET1_R | TCC TGT GAT TGA GGC AGC AG | 108 % |
| TET2_L | TGT CCA GTG AAG GTG CTC AG | 92 % |
| TET2_R | GTT GCT GCA AGT CAG GCA TA | 92 % |
| TET3_v1_L | ATG CCT TCA CTC GCC TCA C | 94 % |
| TET3_v1_R | CAC CCT GTT GCT AGC AGT CT | 94 % |
| TET3_v2_L | AAG CCA AAC ATG GGC ATA AG | 91 % |
| TET3_v2_R | CGC ACC ATC AGC TAC TTT GA | 91 % |
| MeCP2_L | GAC CAG GAC CTC GAC ACA AG | 108 % |
| MeCP2_R | GTG TTG GAG TCC TGG TCT GG | 108 % |
| 18S_RNA_L | GAA CTC ACC GAC ACC AGC A | 93 % |
| 18S_RNA_R | ATC ATC GAC GCT CCT GGA | 93 % |
| ß-actin_L | CTT GCG GAA TCC ACG AGA CC | 92 % |
| ß-actin_R | CCA GGG CTG TGA TCT CCT TCT G | 92 % |
